# Double Insurance for OC: miRNA-Mediated Platinum Resistance and Immune Escape

**DOI:** 10.3389/fimmu.2021.641937

**Published:** 2021-04-01

**Authors:** Xueqin Zou, Yangjing Zhao, Xiuting Liang, Hui Wang, Yanling Zhu, Qixiang Shao

**Affiliations:** ^1^ Reproductive Sciences Institute, Jiangsu University, Zhenjiang, China; ^2^ Department of Immunology, School of Medicine, Jiangsu University, Zhenjiang, China; ^3^ Department of Obstetrics and Gynecology, Xuzhou Hospital Affiliated to Jiangsu University, Xuzhou, China; ^4^ Jiangsu College of Nursing, School of Medical Science and Laboratory Medicine, Huai’an, China

**Keywords:** miRNA, ovarian cancer, platinum, chemoresistance, cisplatin, immune escape

## Abstract

Ovarian cancer (OC) is still the leading cause of death among all gynecological malignancies, despite the recent progress in cancer therapy. Immune escape and drug resistance, especially platinum-based chemotherapy, are significant factors causing disease progression, recurrence and poor prognosis in OC patients. MicroRNAs(miRNAs) are small noncoding RNAs, regulating gene expression at the transcriptional level. Accumulating evidence have indicated their crucial roles in platinum resistance. Importantly, they also act as mediators of tumor immune escape/evasion. In this review, we summarize the recent study of miRNAs involved in platinum resistance of OC and systematically analyses miRNAs involved in the regulation of OC immune escape. Further understanding of miRNAs roles and their possible mechanisms in platinum resistance and tumor escape may open new avenues for improving OC therapy.

## Introduction

Ovarian cancer (OC) is gynecologic malignancy with high mortality rate and is predicted to be the fifth leading cause of female cancer deaths in the United States (1). The high mortality of OC remains a global health problem despite the recent progress achieved in OC treatment. The failure in early diagnosis and drug resistance result in poor prognosis of OC patients (2). Thus, it is urgent to address the underlying mechanisms contributing to poor prognosis and develop more effective therapeutic strategies.

Platinum-based chemotherapies are antitumor agents that are widely used as a first-line clinical therapeutic regimen for OC. Despite a high initial response rate, the majority of advanced stage OC patients will become increasingly resistant to platinum and have a poor prognosis (3). Immune escape/evasion is another critical problem that cannot be ignored in the treatment of OC. OC is capable of create a highly complex and heterogenous ecosystem where anti-tumor immune cells may be hijacked to evade human immune attack (4). Moreover, though immunotherapy has produced promising results in some malignancies, the therapeutic effect of OC patients is not ideal, which has also been mainly attributed to the immune evasion (5).

MicroRNAs (miRNAs) are endogenous non-coding RNAs containing 18 to 25 nucleotides, which play important roles in regulating target gene expression through incompletely pairing to the 3′-untranslational region (3′-UTR), 5′-UTR or even open reading frame and thus causing the degradation of target mRNAs or blocking the translation (6). MiRNAs have differential expression profile in OC and exhibit significant impact on OC occurrence and progression (7). Emerging evidence shed light on the role of miRNAs in platinum resistance in OC and uncover multiple molecular mechanisms of miRNA-based immune escape.

In this review, we summarize the current knowledge of miRNAs involved in platinum chemoresistance and immune escape in OC and discuss their potential applications for improving OC treatment.

## The Role of miRNAs in Platinum Resistance of OC

**Figure 1:**
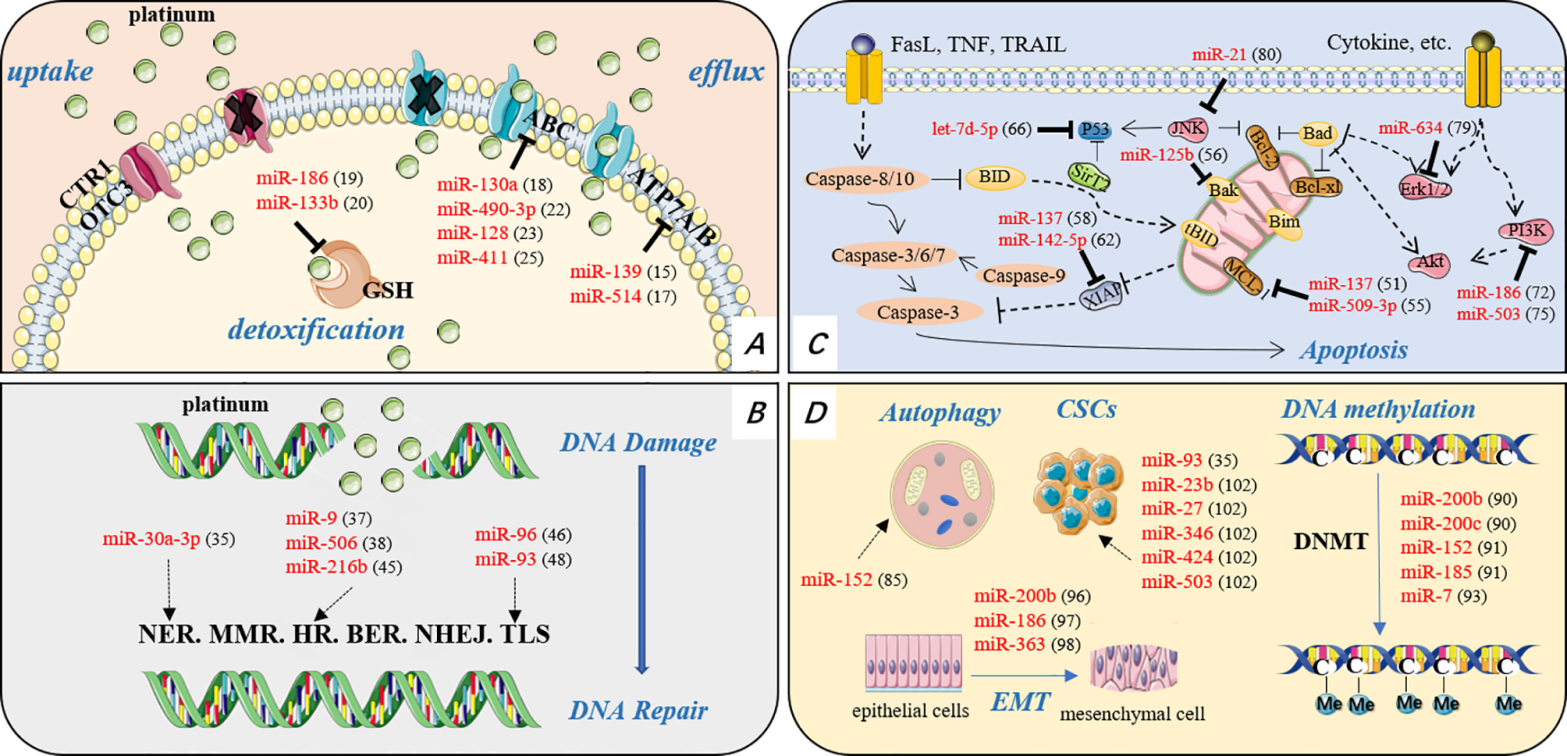
The mechanism of platinum resistance in OC. **(A)** Reduced intracellular accumulation and cytosolic inactivation. **(B)** Increased DNA repair, including NER, MMR, HR, BER, NHEJ, and TLS. **(C)** Alteration of apoptosis related proteins and apoptosis associated pathways causing enhanced resistance to apoptosis. **(D)** Other cellular processes or pathways indirectly reduce platinum sensitivity. CTR1, copper transporter 1; OTC3, organic cation transporter 3; ABC, ATP-binding cassette transporter; ATP7A/7B, P-type ATPase transporter; GSH, glutathione; NER, nucleotide excision repair; MMR, mismatch repair; HR, homologous recombination; BER, base excision repair; NHEJ, non-homologous end joining; TLS, translesion synthesis; EMT, epithelial-mesenchymal transition; DNMT, DNA methyltransferase.

Common mechanisms underlying resistance to platinum-based chemotherapy in OC have been elegantly elucidated by Samuel and colleagues, including reduced intracellular accumulation of platinum and cytosolic inactivation, DNA damage repair, apoptotic pathways abnormality elicited by platinum-mediated DNA damage, and other cellular processes indirectly involved in platinum-elicited signals (8).The aberrant expression and function of miRNAs involved in these mechanisms have been found to be associated with platinum resistance (**Figure 1**).

### Reduced Intracellular Accumulation and Cytosolic Inactivation

#### Defective Drug Uptake

It is widely accepted that platinum is mainly uptaken into cells by passive diffusion and various endocytic routes across plasma membrane. Several transporters are also found to be involved in platinum uptake and efflux, including copper transporter 1 (CTR1), organic cation transporter 2 (OTC2), P-type ATPases (ATP7A/ATP7B) (9). It was demonstrated that Ctr1p mediates cisplatin uptake in yeast and mammals, and cisplatin accumulation was obviously decreased in Ctr1-mutant mouse cell lines compared with wild-type cells (10). Furthermore, OC patients with lower CTR1 expression exhibited increased platinum resistance and shorter survival (11). MiR-130a was reported to target CTR1and induce cisplatin resistance of cervical cancer cell (12). Further research was needed on miRNAs, which affect platinum resistance in OC *via* targeting CTR1.

#### Increased Efflux

Increasing transporter efflux is another reason of decreased intracellular accumulation, in platinum resistance. P-type ATPase transporters and ATP-binding cassette (ABC) transporters are two major transporters involved in platinum efflux (13). Some miRNAs have been shown to affect the chemosensitivity of OC cells *via* regulating these transporters.

ATP7A and ATP7B, which belong to P-type ATPase transporters, are associated with cisplatin resistance in OC cells. ATP7A and ATP7B were reported to mainly localize in the trans-Golgi network in cisplatin-sensitive A2780 cells, while sequestrated in the vesicular structures in cisplatin-resistant cells. This altered localization of P-type ATPase transporters was considered to prevent cisplatin from accumulating in the cytoplasm or nucleus (14). MiR-139 was downregulated in cisplatin-resistant OC tissues (n=14) compared with cisplatin-sensitive tissues (n=23) and was inversely correlated with ATP7A and ATP7B expression. MiR-139 could enhance cisplatin sensitivity in OC cells by suppressing ATP7A and ATP7B expressions (15).

To date, the ABC superfamily contains 49 members and is classified into seven subfamilies (from ABCA to ABCG). ABC transporter-mediated active efflux of cytotoxic compounds, including platinum, across membranes is largely attributed to therapeutic resistance (16). Low expression of miR−514 was reported to be correlated with poor prognosis in OC patients. Moreover, luciferase assay and Western blot analysis confirmed that miR-514 can bind directly to ABC subfamily members, including ABCA1, ABCA10, and ABCF2. Upregulation of miR-514 increases the sensitivity of OC cells to the cytotoxic effect of cisplatin by suppressing ATP binding cassette subfamily expression (17). It was also evidenced that miR-130a could induce OC cells resistance to cisplatin by increasing the mRNA level of MDR1, which was the first identified ABC transporter (18). In addition to regulating ABCB1 protein expression, miRNA can directly target ABCB1. For example, miR-186 directly targeted ABCB1 to sensitize OC cell to cisplatin (19). Similarly, miR-133b increased OC cells sensitivity to cisplatin by inhibiting the expression of ABCB1 and GST-π (20). The nuclear membranous localization of ABCC2, one member of ABC transporter, in OC cells was associated with cisplatin resistance. Moreover, ABCC2 expression was obviously evaluated in the most cisplatin-resistant cell line, A2780RCIS (21). While miR-490-3p could increase the sensitivity of cisplatin in OC cells by inhibiting ABCC2 (22). Besides, ABCC5 and ABCG2, which increased OC chemo-resistance, were also inhibited by miRNAs. Overexpression of miR-128 could reduce the expression of ABCC5 and reverse resistance to cisplatin in OC. Of note, cisplatin combined with miR-128 agomirs inhibited the growth of SKOV3/CP xenografts more effectively than cisplatin alone (23). It has been shown that miR-411 downregulated the expression of ABCG2, causing increased sensitivity of OC cells to cisplatin (24). These miRNAs may act as promising therapeutic targets for the improvement of cisplatin sensitivity in OC (**Table 1**).

**Table 1:** miRNAs mediate platinum resistance *via* regulating intracellular accumulation in OC.

miRNAs	Cell/Tissue	Chemotherapeutic	Target mRNA	Response to treatment	References
miR-139	CAOV3, SNU119, Tissue	Cisplatin	ATP7A/B	↑	(15)
miR-514	OVCA433, SKOV3	Cisplatin	ABCA1, ABCA10, and ABCF2	↑	(17)
miR-130a	A2780	Cisplatin	MDR1, PTEN	↓	(18)
miR-133b	A2780, OVCAR3	Cisplatin, Paclitaxel	MDR1, GST-π	↑	(19)
miR-186	A2780, OVCAR3	Cisplatin, Paclitaxel	ABCB1, GST-π	↑	(20)
miR-490-3p	OVCAR3, SKOV3, Tissue	Cisplatin	ABCC2	↑	(22)
miR-128	OVCAR3, SKOV3, PEO14	Cisplatin	ABCC5, Bmi-1	↑	(23)
miR-411	OVCAR3, SKOV3	Cisplatin	ABCG2	↑	(25)

#### Cytosolic Inactivation

Platinum can also bind to cytosolic nucleophile proteins to be detoxified, including glutathione, methionine, metallothionein, and other cysteine-rich proteins (25). High expression of glutathione S-transferase (GST) was found in a cytotoxic drug-resistant ovarian adenocarcinoma cell line (26). GST-π is an enzyme closely related to drug resistance. GST-π mRNA expression was upregulated after anti–miR-186 transfection in OC cells, which subsequently increases drug resistance (19). MiR-133b also interacts with GST-π and is downregulated in drug-resistant ovarian carcinomas and cell lines. Enhanced expression of miR-133b could effectively sensitize ovarian carcinoma cell lines to chemotherapy drugs (20).

### Increased DNA Repair

Platinum can be activated into hydrated, charged electrophile to bind to DNA and induce DNA damage and cancer cell apoptosis after imported into cytoplasm (27). However, platinum-resistant cells have the ability to tolerate DNA lesions and accelerate DNA repair processes. There are six DNA repair pathways, including direct repair, mismatch repair (MMR), nucleotide excision repair (NER), homologous recombination (HR), base excision repair (BER), and non-homologous end joining (NHEJ). Translesion synthesis (TLS) was considered as a DNA damage tolerance mechanism (28). Increasing evidences demonstrated that miRNAs also influence DNA damage repair processes (**Table 2**). MiR-211 was positively correlated with OC prognosis and could enhance platinum chemosensitivity by blocking the DNA damage response (DDR) (29).

**Table 2:** Increased DNA repair and miRNAs in OC.

miRNAs	Cell/Tissue	Chemotherapy	Target mRNA	Response to treatment	References
miR-211	Tissue	Platinum	DDR	↑	(29)
**NER**					
miR-30a-3p	A2780, IGROV1	Cisplatin	ERCC1, ATP7A	↑	(30)
**HR**					
miR-9	Tissue	Cisplatin	BRCA1	↑	(31)
miR-506	OVCA433, SKOV3, HeyA8	Cisplatin	RAD51	↑	(32)
miR-216b	SKOV3	Cisplatin	PARP1	↑	(33)
**TLS**					
miR-96	U2OS, HeLa, HCC1937, MDA-MB-231	Cisplatin	REV1, RAD51	↑	(34)
miR-93	SKOV3	Cisplatin	Pol η	↑	(35)

#### Mismatch Repair

Mismatch repair (MMR), a highly conservative process, repairs base-base mismatches, insertion and deletion loops that are generated during DNA replication. MMR could recognize mismatch lesions induced by platinum but fail to repair them. MMR appears to be more effective at mediating the cell-cycle and apoptotic responses induced by platinum. MMR deficiency was thus found closely related to platinum resistance in multiple tumor cell lines (57). Aebi et al. have developed a number of different models to explain chemoresistance in MMR-deficient cells. Compared to control cells, cisplatin-resistant OC could induce mutations that functionally alter MMR proteins. More importantly, MMR deficiency contributed to cisplatin resistance in two human tumor cell lines (58). A recent study showed that overexpression of miR-155 significantly down-regulates the core MMR proteins, hMSH2, hMSH6, and hMLH1, inducing a mutator phenotype and MSI, which is a signature of MMR defects. Although further study is needed, this study strongly supports a role for miRNAs in the non-Mendelian regulation of MMR genes and MMR-deficient related platinum resistance (59).

#### Nucleotide Excision Repair

Increased nucleotide excision repair (NER) was shown in cisplatin-resistant ovarian tumor cells. ERCC1–XPF, a NER protein responsible for cutting the strand on the 5′ side of the damage endonuclease, may be a determinant of increased NER in cisplatin-resistant model (60). To determine the impact of ERCC1, a component of ERCC1–XPF, in cisplatin drug resistance, Selvakumaran et al. (61) established stable OC lines expressing antisense ERCC1 and found that cisplatin sensitivity of these cell lines were enhanced. ERCC1 mRNA is a direct target of miR-30a-3p, which notably increased the DNA damage and intracellular cisplatin accumulation in ovarian carcinoma cells. Sulforaphane, one of the most available phytochemicals in cruciferous vegetable, could increase miR-30a-3p expression and repress ERCC1 and ATP7A, contributing to enhanced cisplatin sensitivity in OC cells. In other words, the impaired DNA repair mediated by ERCC1 could be corrected by miR-30a-3p combined with sulforaphane in cisplatin-resistant OC cells (30).

#### Homologous Recombination

Double-strand breaks (DSBs) are repaired by HR during the S and G2 phases of the cell cycle. The process of homologous recombination (HR) mainly includes three parts: damage recognition by the kinases ATM and ataxia telangiectasia and Rad3-related, signal transduction by CHK2 and BRCA1, and initiation of repair by BRCA2 and RAD51 (62). MiRNA can regulate cisplatin sensitivity in OC by targeting important component of HR. One study demonstrated that 3′-UTR of BRCA1 mRNA is a direct functional target of miR-9, which is associated with cisplatin sensitivity and good prognosis of OC patients. MiR-9 can increase the sensitivity of OC to cisplatin and promote DNA damage *via* inhibiting BRCA1. Therefore, miR-9 may serve as a promising therapeutic target for OC patients who exhibit resistance to cisplatin (31). Analyzing the Cancer Genome Atlas (TCGA) database network for high-grade serous ovarian cancer (HGSOC), Liu found that miR-506 expression was associated with an increased response to therapy and prolonged progression-free survival (PFS) and overall survival (OS). Further results have indicated that RAD51 is a direct target of miR-506. MiR-506 can enhance the response to cisplatin through targeting RAD51 and suppressing homologous recombination in OC cell lines (32). In addition to the dysregulation of HR components, HR deficiency also related to platinum resistance in OC. About half of serous ovarian cancer (SOC) have defects in homologous recombination, while BRCA1/2 were mutated in 22% of tumors (63). Although BRCA1/2-mutated OC are initially sensitive to platinum, such cancer still later develops cisplatin resistance. Secondary intragenic mutations in BRCA2 may be one reason for acquired drug resistance in BRCA2-mutated cancers (64). The mechanisms of resistance in BRCA2-mutated cancers are much more than those, emerging evidence indicated that miRNAs are also involved. Choi has uncovered that miR-622 could induce resistance to platinum in BRCA1-mutant HGSOCs by targeting the Ku complex and restored HR-mediated double-strand breaks repair (65). Similarly, miR-493-5p also mediated platinum resistance in BRCA2 mutant carcinomas. But miR-493-5p reduced genome stability rather than restore HR (66). Furthermore, BRCA2-deficient cells appear to rely on specific repair pathways, such as BER (67). PARP1 is involved in BER which mediates the repair of DNA single-strand breaks (68). Liu has revealed that PARP1 is a direct target of MiR-216b, which is downregulated in cisplatin-resistant OC cells. Overexpression of MiR-216b could increase cisplatin sensitivity in OC cells by targeting PARP1 (33).

#### Translesion Synthesis

Translesion synthesis (TLS) allows to synthesize DNA *via* DNA lesions and is easy to error, so it is considered as a DNA damage tolerance mechanism (28). It has been previously shown that suppression of TLS improves therapy sensibility and prevents tumor chemoresistance. As an essential TLS scaffold protein and dCMP transferase, Rev1 plays a key role in preventing cisplatin cytotoxicity and DNA damage-induced mutagenesis. Upregulated expression of miR-96 augmented cisplatin sensitivity by repressing REV1 in OC cells (34, 69). Besides, miR-93 increases the efficacy of cisplatin treatment *via* negatively regulating TLS DNA polymerase η (Pol η) in OC stem cells (CSCs) (35).

### MiRNAs and Apoptosis-Associated Pathway Inactivation

Once DNA damage caused by platinum failed to be repaired, tumor cells undergo programmed cell death. Inactivation of apoptosis pathway to escape from platinum-induced apoptosis in tumor cells is one of the important mechanisms of platinum resistance. A number of miRNAs have been shown to be involved in regulating apoptosis-related proteins (**Table 3**) and pathways (**Table 4**).

**Table 3:** MiRNAs that modulate chemoresistance through apoptosis related proteins in OC.

miRNAs	Cell/tissue	Chemotherapy	Target mRNA	Response to treatment	References
**BCL-2 family members**					
miR-137	OVCAR3	Cisplatin	MCL1	↑	(36)
miR-17~92 cluster	A2780, OVCAR3, SKOV3	Cisplatin	BCL2	↑	(37)
miR−335- 5p	A2780, OVCAR3, OV90	Cisplatin	BCL2L2	↑	(38)
miR−146a−5p	OVCAR3, SKOV3	Cisplatin	BCL2L2, XIAP, BIRC2, BIRC5	↑	(39)
miR-509-3p	OVCAR3, SKOV3	Cisplatin	BCL2, BCL2L2, MCL1	↑	(40)
miR-125b	OV2008, C13*	Cisplatin	Bak1	↓	(41)
**XIAP**					
miR-137	A2780, SKOV3	Cisplatin	XIAP	↑	(42)
miR-130a	A2780	Cisplatin	XIAP	↑	(43)
miR-519d	A2780, OVCAR3, SKOV3	Cisplatin	XIAP	↑	(44)
miR-149	SKOV−3, HO8910, ES2	Cisplatin	XIAP	↑	(45)
miR-142-5p	OVCAR3, SKOV3	Cisplatin	XIAP	↑	(46)
**P53**					
let-7d-5p	A2780, OVCAR3, SKOV3	Cisplatin	HMGA1	↑	(47)

**Table 4:** Inactivation of apoptosis related pathway and miRNAs in OC.

miRNAs	Cell/tissue	Chemotherapy	Target mRNA	Response to treatment	Reference
**PI3K/Akt pathway**					
miR-221/222	A2780	Cisplatin	PTEN	↓	(48)
miR-216a	OVCA433, SKOV3	Cisplatin	PTEN	↓	(49)
miR-186	A2780, SKOV3	Cisplatin	PTEN, APAF1	↑	(50)
miR-186	A2780, SKOV3	Cisplatin	PIK3R3	↓	(50)
miR-34c	A2780, SKOV3	Cisplatin	MET	↑	(51)
miR-124-3p.1	A2780, SKOV3	Carboplatin	CAV1	↑	(52)
miR-503	SKOV3	Cisplatin	PI3K, p85	↑	(53)
**MAPK pathway**					
miR-330-5p	Caov3, SKOV3	Cisplatin	S100A7	↑	(54)
miR-634	A2780, OV56, OAW42	Cisplatin	Ras-MAPK pathway	↑	(55)
miR-21	SKOV3ip1, HEYA8, A2780, CP20	Cisplatin	JNK-1/c-Jun	↓	(56)

#### Apoptosis-Associated Proteins

##### BCL-2 Family Members

Bcl-2 family in mammals contains 20 proteins and is divided into two categories. The anti-apoptotic proteins include Bcl-2, Bcl-XL, Bcl-W, McL-1, etc. On the contrary, the pro-apoptotic proteins include Bax, Bak, Bok, and their subsets, such as Bad, Bim, Bid, Noxa, Puma, Bik/Blk, Bmf, Hrk/DP5, Beclin-1, and Mule (70, 71). MiRNAs could mediate drug resistance *via* regulating apoptosis by targeting pro-apoptotic genes or anti-apoptotic genes. Chen has found that miR-137 promoted cisplatin-induced apoptosis *via* downregulating MCL1 in OC cells (36). BCL-2 is identified as a direct target of the miR-17–92 clusters, which promotes cisplatin-induced apoptosis in OC cells (37). MiR-335-5p, which was down-regulated in cisplatin-resistant A2780 cells, enhanced cisplatin-induced cell apoptosis by targeting BCL2L2 (38). Additionally, miR−146a−5p downregulates several anti−apoptotic genes, including XIAP, BCL2L2, and BIRC5. MiR-146a-5p can effectively accelerate apoptosis by sensitizing Epithelial ovarian cancer (EOC) cells to cisplatin, which can be rescued by XIAP overexpression (39). Similarly, miR-509-3p can decrease the expressions of BCL2, BCL2L2, and MCL1 and sensitize OC cells to cisplatin treatment (40). On the contrary, miR-125b repressed Bak1 expression, which plays a critical role in cisplatin-induced apoptosis to promote cisplatin resistance in OC (41).

##### X-Linked Inhibitor of Apoptosis Protein (XIAP)

As a member of apoptosis protein family inhibitors, XIAP can directly inhibit several caspases in the central parts of apoptosis pathways (72). MiRNA-137 promotes apoptosis by decreasing XIAP protein levels (42). Similarly, miR-130a, miR-519d, and miR-149 can directly bind the 3′-UTR of XIAP and enhance cisplatin-induced apoptosis (43–45). MiR-142-5p can regulate multiple anti-apoptotic genes including XIAP and can be considered as potential treatment targets and theragnostic panel in OC (46).

##### p53

Wild type p53 protein mediates the inhibition of DNA synthesis that follows DNA damage (73), so the deactivation of p53 is associated with chemoresistance in OC. Most OC is characterized by TP53 mutations, genetic mutation is not the only cause of p53 inactivation (74). Recent studies have demonstrated that miRNAs are involved in the regulation of p53 protein in an indirect way. In SOC, low expression of miR-31 targeting CDKN2A is associated with defects in the p53 pathway. CDKN2A encodes the tumor suppressor proteins p14^arf^ which sequesters MDM2, a potent inhibitor of p53 (75). It was demonstrated that miRNA let−7d−5p was responsible for promoting OC cell apoptosis and increasing chemosensitivity by regulating the p53 signaling pathway *via* HMGA1 (47). These findings favor the application of miRNAs in OC with deficiency of p53 activity.

#### Apoptosis-Related Pathways Pathway

##### PI3K/Akt Pathway

The PI3K/AKT pathway is the hub of a variety of signal pathways including apoptosis. Ersahin constructed a comprehensive PI3K/AKT/mTOR signaling pathway consisted of 254 components and 478 links from 498 peer reviewed literature. The regulatory network is clearly shown that PI3K/AKT pathway is regulated by a variety of upstream regulatory proteins, such as PTEN, PI3K, and RTKs, and involves in multiple pathways through regulating many downstream effectors, such as GSK-3β, FOXO, and MDM2 (76). So, if miRNA can control regulatory proteins in the upstream of the PI3K/AKT pathway, it is highly likely to affect downstream effectors through this pathway and thus regulate cell cycle and apoptosis. For example, miR-221/222 were found to induce cisplatin resistance by targeting PTEN-mediated PI3K/Akt pathway (48). PTEN, an important tumor suppressor, antagonizes PI3K activity *via* regulating the cellular level of PIP3 (77). Moreover, it has been reported that inhibition of PI3K/Akt/mTOR signaling pathway enhances cisplatin sensitivity in the drug-resistant human OC cells SKOV3/DDP OC cell line (78). Analogously, miR-216a also directly target PTEN and promote cisplatin resistance of OC cells. Although the report has been declared that STAT3 is a regulator of miR-216a, the miR-216a/PTEN/PI3K/Akt axis need to be explored in the future (49). It is worth mentioning that miR-186 play the bidirectional regulatory role of cisplatin sensitivity in OC. MiR-186 inhibited the expression of PTEN and PIK3R3 dose-dependently, which are play a completely opposite role in the AKT pathway. PTEN mediated increasing cisplatin sensitivity of OC cells when miR-186 is at low concentration, while PIK3R3 decreased the cisplatin sensitivity under the context of high concentration of miR-186 (50).

In addition to PTEN, miRNAs can also regulate other proteins in the upstream of the AKT pathway. MiR-34c inhibited the phosphorylation of PI3K and AKT and thus activate Bad through targeting MET. Since Bad (BCL2-associated agonist of cell death) is a pro-apoptotic protein, it can sensitize OC cells to cisplatin-induced mitochondrial apoptosis (51). Likewise, miR-124-3p.1 may sensitize OC cells to mitochondrial apoptosis *via* the CAV1/AKT/Bad pathway (52). MiR-503 can regulate PI3K p85 to reduce the cisplatin resistance of OC cells by the PI3K/Akt signaling pathway (53). These data fully illustrated the diverse roles of miRNA in the PI3K/Akt signaling pathway.

##### MAPK Pathway

MAPK pathway plays a critical role in cisplatin-induced apoptosis. There are three major MAPK subfamilies described in mammalian: the p38 MAPK, Jun kinase (JNK), and ERK pathway. Mansouri compared the cisplatin-induced activation of these three MAPKs between cisplatin-sensitive and cisplatin-resistant human OC cell line. Results have uncovered that cisplatin-induced apoptosis depends on c-Jun, especially the duration of JNK activation. Moreover, the JNK/p38 MAPK pathways led to sensitization to apoptosis *via* reactivating FasL expression in resistant cells (79). ERK activation has also been demonstrated to be necessary for cisplatin-induced apoptosis (80). Regulation of the three MAPK subfamilies by miRNAs can also affect resistance in OC. For example, S100A7, a target of miR-330-5p, increased the expression of active p38, JNK, and ERK in EOC. This suggests that miR-330-5p can be a possible target for increasing sensitivity to cisplatin (54). Overexpressed miR-634 directly repressed the Ras-MAPK pathway components GRB2, ERK2, and RSK2. MiR-634 not only resensitized resistant OC cell lines to cisplatin but also to carboplatin and doxorubicin (55). In addition, some miRNAs reduced chemosensitivity in OC cells through the downstream of MAPK pathway. Echevarría-Vargas has reported that the c-Jun binding to miR-21 gene promoter regions, while the expression of miR-21 is reduced after blocking the JNK-1 which participate in activating phosphorylation of c-Jun. The JNK-1/c-Jun/miR-21 pathway is believed to increase the cisplatin resistance of OC cells (56).

#### Indirect Mechanisms Associated With Platinum Resistance

Accumulating evidences suggest that miRNAs were also involved in drug resistance of OC *via* some indirect mechanisms.

##### Autophagy

Autophagy is a highly conserved pathway which engulfs cytoplasmic components and delivers them to the lysosome for degradation. It can be a double−edged sword in the development of tumor. On the one hand, autophagy removes damaged cells and recycling the raw material in normal cells, but on the other hand, autophagy may be a survival mechanism that promotes chemoresistance following chemotherapy (81, 82). It has not been fully revealed how autophagy protects tumor cells from death mediated by chemotherapy. Wang found that cisplatin treatment might induce autophagy through activating ERK and promoting resistance in OC cells. So, inhibition of autophagy has the potential to improve chemotherapeutic resistance (83). Overexpression of miR-29c-3p may be a useful therapeutic strategy to inhibit autophagy and DDP resistance partly *via* downregulating FOXP1/ATG14 pathway. ATG14, belonging to autophagy-related (ATG) proteins, plays an important role in the initiation steps of autophagy (84). It is also a target of miR-152, which can resensitize cisplatin-resistant in OC cells by reducing cisplatin-induced autophagy. Furthermore, miR-152 is regulated by EGR1. Both activation of EGR1 and upregulation of miR-152 can increase chemosensitivity in OC (85). Another example of miRNA regulation of autophagy is miR-204, which inhibited autophagy in OC cells and overcome cisplatin-resistance *via* targeting LC3B (86). These findings only reveal partial role of miRNAs in regulation of autophagy in cisplatin-resistant cells. More evidences need to be uncovered and explored.

##### Epigenetics

Epigenetics refers to variability in gene expression, heritable through mitosis and potentially meiosis, without any underlying modification in the actual genetic sequence. The most studied epigenetic machineries are DNA methylation, histone modifications, and small or long noncoding RNAs (lncRNAs) (87). To investigate DNA methylation associated with platinum resistance, the researchers analyze the global CGI methylation and mRNA expression of drug-sensitive and -resistant A2780 EOC cells after treating with increasing concentrations of cisplatin. Results showed that hypermethylated CGIs are related to increased drug resistance (88). In addition, low-dose decitabine, a kind of hypomethylating agents, restored sensitivity to carboplatin in platinum-resistant OC patients (89). Recent studies have also shown that miRNA can regulate DNA methylation and improve chemotherapeutic efficacy. In OC, miR−200b and miR−200c enhance cisplatin sensitivity *via* targeting DNA methyltransferases (90). Similarly, miR-152 and miR-185 were also identified as a negative regulator of DNA methyltransferase 1 (DNMT1), which mediates DNA methylation (91). Expression of some miRNAs is regulated by genetic or epigenetic events, and these miRNAs are involved in platinum response of OC. Deng has found that the miR-199a promoter was hypermethylated in OC cells but not in normal ovarian epithelial cells. Overexpression of miR-199a enhanced cisplatin resistance through inhibiting DDR1 expression (92). MiR-7, which presented specific methylation in resistant cell lines, reduces cell sensitivity to cisplatin by targeting MAFG (93). Additionally, let-7e are down-regulated in cisplatin-resistant human EOC cell line A2780/CP. Further study demonstrated that DNA hypermethylation is the cause of let-7e silencing in OC (94).

##### Epithelial-Mesenchymal Transition

Strong evidences show that epithelial-mesenchymal transition (EMT) is associated with resistance to platinum-based chemotherapy in EOC (95). MiRNAs may partially mediate platinum resistance or sensitivity through regulating EMT. Drug-resistant ovarian cell lines expressed EMT phenotype, while transfection with all miR-200 family members generally induced morphological hallmarks of mesenchymal-epithelial transition (MET). It is worth mentioning that each individual member of miR-200 family exists significant differences in the regulation of chemotherapy sensitivity. Cells transfected with miR-200b were significantly more sensitive to cisplatin than those transfected with miR-429 (96). The important components, such as Snail, Slug, Twist in the occurrence of EMT may be direct targets of miRNAs. For example, Twist1, which was negatively regulated by miR-186, induced EMT and cisplatin resistance in EOC (97). Cao has found that miR-363 inhibits cisplatin chemoresistance of EOC, with a decreased expression of Snail, which plays an important role in initiating of EMT (98).

##### Cancer Stem Cells

OC is considered as a kind of stem cell–related disease, which originates from the fallopian tube and ovarian surface epithelium (99, 100). Cancer stem cells (CSCs) are characterized by enhanced tumorigenicity and chemo-resistance. Srivastava has found that ovarian CSCs may have intrinsically enhanced TLS through increasing the expression of Pol η and which allows ovarian CSCs to survive cisplatin treatment. What is more, further study has shown that miR-93 inhibited the expression of Pol η. It suggests that miR-93 is able to be a therapeutic target that can enhance the treatment of cisplatin (35). The characteristics of CSCs are majorly similar to normal stem/progenitor cells, so the search for unique markers of CSCs is necessary (101). There is evidence that ALDH1 is a useful marker for enriching ovarian CSCs. MiR-23b, miR-27a, miR-27b, miR-346, miR-424, and miR-503, which overexpressed in ALDH1+ cells, are significantly associated with chemoresistance and tumor progression in OC (102). In addition, side population (SP) cells are putative CSCs, which sorting based on ABC transporter–mediated efflux of the Hoechst 33342 dye (103). By using a quantitative PCR array, Wei found that miR-551b was upregulated in the SP cells isolated from an ascite-derived OC cell. Further assays uncovered that miR-551b promoted chemoresistance through suppressing the expression of Foxo3 and TRIM31 in OC (104).

## MiRNAs Modulating Immune Escape/Evasion in OC

**Figure 2:**
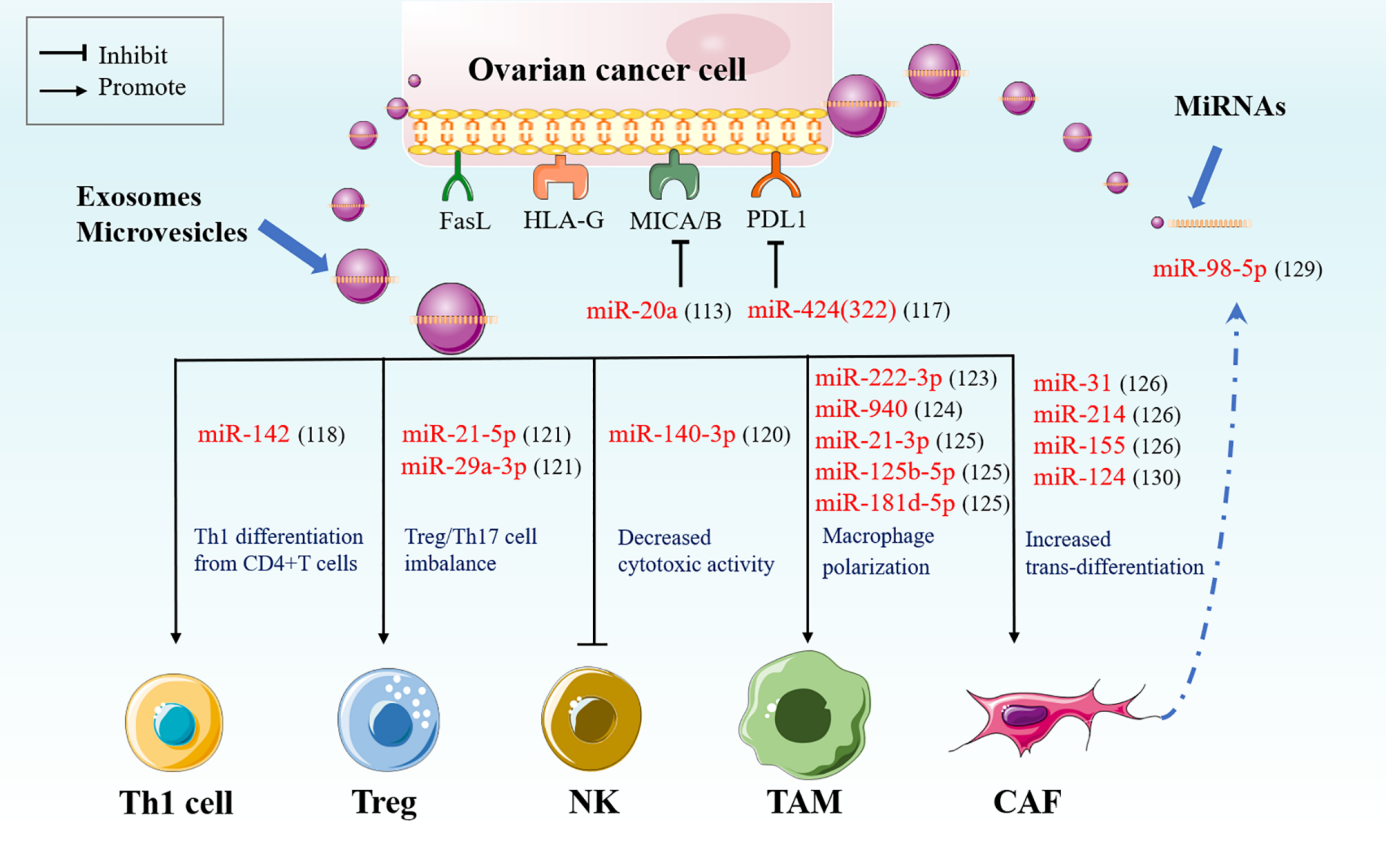
miRNA mediated immune escape in OC. Immunological miRNA can suppress MHC class I chain-related molecules A and B (MICA/B) and immune checkpoint PDL1. OC cells-derived miRNAs can regulate immune evasion *via* exosomes or vehicles. OC cells-derived miRNAs promote Th1 differentiation from CD4+T cells, blocking NK cells cytotoxicity, inducing the polarization of macrophages toward M2-like phenotype, as well as reprograming normal fibroblasts to CAFs. Moreover, CAFs can secrete exosomal miRNAs to promote the progression of OC. MICA/B, MHC class I chain-related molecules A and B; NK, natural killer cells; TAMs, tumor-associated macrophages; CAFs, cancer-associated fibroblasts.

Apart from the role of miRNAs in platinum resistance of OC, miRNAs associated with immune response or immune regulation have recently also attracted more attention in cancer. These miRNAs are involved in tumor escape/evasion *via* regulating the immunogenicity of tumors and antitumor immune responses (105). Fundamental progress has been made to understand the molecular mechanisms underlying immune escape by tumors, which contributes to a new dimension in understanding of tumor development and progression. These mechanisms mainly include either defects or decreases in classic HLA class I antigens, components of the antigen processing machinery (APM) and the interferon (IFN) signaling pathway, or upregulation of non-classical HLA class I antigens and negative immune checkpoints. Besides, immune escape is associated with the low level of CD8+ and CD4+ T cells, dendritic cells (DCs), as well as natural killer (NK) cells, which mediate anti-tumor immune responses, increased immune suppressive cells containing regulatory T cells (Treg), tumorassociated macrophages (TAMs), tumor-associated neutrophils (TANs), and myeloid-derived suppressor cells (MDSCs). The altered tumor and immune cell metabolism and the shaping of tumor microenvironment also contributed to immune escape. Moreover, tumor cells can release immune-suppressive cytokines (such as TGF-β, IL-10, etc.) and exosomes (containing miRNAs, lncRNAs, proteins, and so on) to affect anti-tumor immune responses (106). A number of studies have declared that miRNAs also affecting OC immune escape *via* some mechanisms above and are described in detail below (**Figure 2**).

### The Effect of miRNAs on OC Antigen Processing and Presentation

#### HLA-G and MHC Class I–Related Proteins

HLA-G is a class Ib HLA molecule, which was initially found at the maternal-fetal interface and plays a key role during pregnancy by maintaining maternal-fetal tolerance (107). While tumors are capable of exploiting HLA-G to promote immune escape *via* suppressing the cytotoxic activities of effector cells, such as CTLs and NKs (108). Lin et al. analyzed HLA-G expression in 33 primary ovarian carcinoma tissues and found that HLA-G expression was detected in 22/33 (66.7%) primary tumor tissues while it was absent in normal ovarian tissues (P<0.01). Further functional study indicated that HLA-G could inhibit NK cell cytotoxicity and thus assist ovarian carcinomas to escape from human immune surveillance (109). HLA-G–related miRNAs get increased attention in recent years. There were miRNA binding sites, also the potential targets of miR-148a, miR-148b, and miR-152 at the 3′-UTR region of HLA-G (107). MiR-148a, miR-148b, and miR-152 were subsequently demonstrated that could downregulate HLA-G expression in other tumors (110), while the role for miRNAs in the regulation of HLA-G in OC remains to be revealed.

MHC class I chain-related molecules A and B (MICA/B) and UL-16 binding proteins (ULBPs) are the ligands of NKG2D, while NKG2D expressed on NK cells, CD8+ cytotoxic T cells (CTL) and TCR γδ-T cells (111). NKG2D signaling plays a critical role in cancer immunosurveillance *via* activating NK cells and T cells, while tumors can develop mechanisms to overcome the NKG2D-mediated immune response and induce immune escape (112). MICA/B suppression by miRNAs is one of the strategies by which OC cells escape immunosurveillance. It was found that miR-20a, which is correlated with OC progression, directly binds the MICA/B 3′-UTR and reduces NKG2D-mediated killing. More importantly, miR-20a are found to mediate immune evasion *in vivo (* 113). In addition, high expression of ULBP2 is reported to be correlated with poor prognoses for OC patients and may related to the functional inhibition of CD8+T cells, while ULBP2 expression has been suggested to be regulated by miR34a and miR34c (111, 112).

#### Immune Checkpoint Proteins

Immune checkpoint proteins (ICPs) are regulators of immune system and divided into two parts according to their effects on T or B cells: co-stimulatory proteins (CD28, ICOS, B7H2, B7H3, CD27, CD70, CD40, and CD40L) and co-inhibitory proteins (PD-1, PD-L1, CTLA-4, and B7-H4), also the negative ICPs (114). The dysfunction of miRNAs which regulate ICPs seems to be one of the important reasons for immune escape in OC. Considering B7H3 is aberrantly overexpressed in many types of cancer and associated with a poor clinical prognosis, researchers revealed that 17 common miRNAs potentially influence B7H3 mRNA through meta-analysis of miRNA database. Among these miRNAs, low miR-187 and miR-489 expression was associated with poor prognosis of OC, with the analysis of the TCGA OC data set. Future studies will provide further insights into B7H3-related miRNA in ovarian carcinoma (115).

Programmed death-ligand 1 (PD-L1), an inhibitory molecule expressed by cancer cells, which assist tumor cells to escape the host immune attack in the tumor micro-environment are found to be evaluated after treating with cisplatin in cisplatin-resistant OC cells. Further study declared that miR-145 can repress PD-L1 expression *via* targeting c-Myc. Thereby cisplatin mediated T lymphocytes dysfunction through miR-145/c-Myc/PD-L1 axis (116). Similarly, Xu et al. have found that miR-424(322), which correlated with the progression-free survival of OC patients, activates CTLs and reduces regulatory cytokine secretions. Mechanistic investigations showed that miR-424(322) negative regulates the PD-L1/PD-1 and CD80/CTLA-4 pathways in chemo-resistant OC (117).

### Cancer Cell-Derived miRNAs and Its Role in Modulation of Antitumor Immune Cells

MiRNAs functions not only within cells, but also through microvesicles and exosomes. Tumor-derived miRNAs can interact with immune cells with the assistance of microvesicles and exosomes, so they are able to promote the immune escape/evasion of cancer cells by either directly inhibiting immune cells such as T cells, DCs, and NK cells or inducing immune-suppressive cells like MDSCs, Tregs, and TAMs (105). Advanced dates point out that miRNAs are capable to regulate immune cells in OC, thereby facilitating tumor immune escape.

#### Lymphocytes

A recent study described that the IFN-level and Th1/CD4^+^T cells percentage were increased in CD4^+^T cells treated with artesunate, but rescued by miR-142 inhibitor. Further investigation found that artesunate promotes Th1 differentiation from CD4+T cells and enhance the pro-apoptotic effects of Th1 cells in OC *via* the miR-142/Sirt1 pathway (118). NK cells are the prototype innate lymphoid cells with enhanced cytolytic function which handles immune surveillance against cancer (119). So, the decreased natural killer cytotoxicity seems to be a cause for tumor escape. It has been reported that, miR-140-3p blocked NK cells cytotoxicity in OC *via* mediating MAPK1, which suggest a new approach to improve NK cells function for OC (120). In addition, miR-29a-3p and miR-21-5p in exosomes are found to alter the ratio of Treg to Th17 cells. A luciferase assay revealed that miR-29a-3p and miR-21-5p directly target the STAT3 3′-UTR and then enhanced the growth and metastasis of OC cells *in vivo* (121).

#### Tumor-Associated Macrophages

Macrophages are heterogeneous and contain two distinct subsets: M1 macrophage which are pro-inflammatory, and M2 macrophage with pro-inflammatory function. In general, tumor-associated macrophages (TAMs) are more resemble to M2 phenotypes, which promote tumor in different aspects (122). Recently, several studies have shown that cancer-derived exosomal miRNA are a pivotal factor accounting for the modulation of TAMs in OC. MiR-222-3p, which is enriched in EOC-derived exosomes, could promote macrophage polarization and differentiation to M2 phenotypes by regulating the SOCS3/STAT3 pathway *in vitro* and *in vivo (* 123). Interestingly, the expression of miR−940 was both increased in EOC cells and EOC−derived exosomes under the condition of hypoxia. Further assays indicated that miR-940 induce M2-phenotype macrophages and thus promote migration of OC cells (124). Similarly, miR-21–3p, miR-125 b-5p, and miR-181d-5p in exosomes derived from hypoxic EOC cells, affected macrophage M2 polarization by regulating the SOCS4/5/STAT3 pathway (125). These exosomes and associated miRNAs provide novel targets for the treatments of EOC.

#### Cancer-Associated Fibroblasts

Cancer-associated fibroblasts (CAFs), the major constituent of the tumor stroma, are proposed to play critical roles in cancer progression (126). Moreover, CAFs also exhibit positive correlation to the invasion and metastasis of EOC (127). Previous study indicated that the crosstalk between miRNAs, tumor cells and fibroblasts is proposed to play critical roles in ovarian carcinogenesis. Trying to mimic a natural condition in OC, researchers co-cultured SKOV-3 cancer cells with human normal fibroblasts derived from primary culture (FP-96). The results uncovered that α-SMA, the common expressed marker in CAFs was induced and expressed in FP-96 fibroblasts under the co-culture system condition. Subsequently, α-SMA–expressing fibroblasts induced the downregulation of tumor suppressor miR-29b expression in SKOV-3 cells and increased migration of SKOV-3 cells. These data suggest that CAFs regulate miRNAs expression possibly in OC, although it has not been fully confirmed (128). Another investigation has shown that CAFs are able to secrete exosomal miR-98-5p which promote cisplatin resistance in OC by downregulating CDKN1A (129). CAFs can secrete exosomal miRNAs under the tumor microenvironment, thereby modulating the expression of genes involved in tumor progression. Conversely, OC cells‐derived miRNAs were involved in promoting the transdifferentiation of normal fibroblasts to CAFs. For example, it was found that OC cells reprogram fibroblasts to become CAFs *via* downregulating miR-31 and miR-214 or upregulating miR-155 (126). Besides, Zhang et al. observed that miR‐124 which secreted by human ovarian surface epithelial cells is involved in reprograming normal fibroblasts to CAFs, thereby triggering the tumor progression (130).

## MiRNA−Related Therapeutic Strategies

Alteration in miRNA expression profile plays a crucial role in carcinogenesis. Overexpressed oncomiRNAs act as oncogenes to induce drug resistance, assist tumor escape, and so on, while oncomiRNAs with lower expression or anti-oncomiRNAs with higher expression act as tumor suppressors to improve chemotherapy sensibility, prevent tumor evasion, etc. It is promising to develop diagnostic markers and therapeutic strategy based on these abnormally expressed miRNAs. However, it is a challenge to select the suitable miRNAs from a large amount of candidate miRNAs.

At present, miRNA-based therapy is realized by artificially modulating the expression of miRNAs. Silencing of aberrant miRNAs can be achieved by the synthetic analogs of small RNA molecules termed “antagomirs” or locked nucleic acid (LNA), while miRNA mimics and modified miRNAs are often used to rescue normal levels of miRNAs that are silenced in cancer cells (131). MRX34, miR−34 mimics, is the first miRNA therapeutics for cancer and achieved phase I clinical trial in several solid and hematological malignancies (132). Since then, numerous preclinical studies involving miRNA therapeutics have been conducted and partial miRNA therapeutics have moved through the clinical development process (131). However, it is still a long way to go before safely and effectively applying to clinic, miRNA therapeutics have extensive application prospects in anti-tumor therapy.

For efficient, miRNA therapeutics need to be transported to the cell *via* an appropriate delivery system. Artificial engineered nanoparticles are designed to delivery miRNA with higher stability and less toxicity. For example, Javanmardi et al. established a new plasmid delivery system, PEG2k-CMPEI-ss, which can deliver anti-miR-21 to OC cells, thereby increasing sensitivity to cisplatin (133). Moreover, accumulating evidence has demonstrated that exosomes, natural physiological nanovesicles, are useful carriers for miRNAs delivery. It was documented that the engineered exosome-based 5-FU and anti-miR-21 co-delivery system could efficiently reverse 5-FU resistance in colon cancer. Moreover, acute toxicity was not detected in mice treated with engineered exosome packaging 5-FU and anti-miR-21 (134). So far, research on exploring effective miRNAs delivery tools is still ongoing.

## Discussions

OC is the most lethal gynecological malignancy across the world. Platinum-based chemotherapy resistance decreases anticancer efficacy of drugs in OC, on the other hand, tumor exploits various mechanisms to escape the attack of human immune cells. Platinum resistance and immune escape provides double insurance for OC, the exploration of the tolerogenic pathways utilized by malignant tumors and development of effective combination therapy are urgently needed. The discovery of miRNAs has deepened our understanding of human cancers.

MiRNAs have been documented to be involved in various biological processes to regulate chemoresistance in OC. The intracellular accumulation of platinum is partially regulated by miRNAs before entering nucleus to induce DNA damage, which has not yet been entirely elucidated. MiRNAs are also involved in DNA damage repairing process, such as HR, MMR and NER. Due to the wide defections of homologous recombination in OC patients, much attention has been paid on HR-associated miRNAs in platinum-resistant OC cells. Generally, most miRNAs affect drug resistance by targeting genes in platinum-induced apoptosis process. MiRNAs also participate in many other indirect mechanisms to regulate platinum resistance, such as regulating in the autophagy, epigenetics, EMT and CSCs. The research on mechanisms of miRNA-related platinum resistance offers promising access to miRNA-based therapy in OC.

In recent years, miRNAs, associated with immune response or regulation, provide new insights into the mechanisms of tumor growth and progression. Tumor derived miRNA interact with immune cells and thus evading from surveillance of immune system. It is an appealing idea to target these miRNAs to overcome tumor immune escape and enhance anti-tumor immune response. As a new field in OC, the immune response associated miRNAs have not been revealed in depth, but it cannot be ignored their clinical value.

In summary, miRNA-based therapy may offer new opportunities for OC patients by overcoming the platinum resistance and immune escape, and research on miRNAs deserves to receive much attention.

## Author Contributions

XL, HW and YZ give the suggestion about the part of miRNAs modulating immune escape/evasion in OC and collected and summarized relevant studies when the manuscript was reversed. HW and YZ were the funding recipients. All authors contributed to the article and approved the submitted version.

## Funding

The present study was supported by grants from National Natural Science Foundation of China (grant nos. 81671541, 81273202, 81701545 and 82071738) and Primary Research & Development Plan (Social Development) of Xuzhou City (KC19147). We also would like to thank Miaomiao Jia for reading the manuscript.

## Conflict of Interest

The authors declare that the research was conducted in the absence of any commercial or financial relationships that could be construed as a potential conflict of interest.
